# Prevalence and incidence of visual impairment in patients with proliferative diabetic retinopathy in India

**DOI:** 10.1038/s41598-020-67350-6

**Published:** 2020-06-29

**Authors:** Rehana Khan, Shruti Chandra, Ramachandran Rajalakshmi, Padmaja Kumari Rani, Giridhar Anantharaman, Alok Sen, Abhishek Desai, Rupak Roy, Sundaram Natarajan, Lanin Chen, Gajendra Chawla, Umesh Chandra Behera, Lingam Gopal, Sarega Gurudas, Sobha Sivaprasad, Rajiv Raman

**Affiliations:** 1 Shri Bhagwan Mahavir Vitreoretinal Services, Sankara Nethralaya, 18 College Road, Chennai, Tamil Nadu 600 006 India; 2 NIHR Moorfields Biomedical Research Centre, London and University College London, London, EC1V 2PD UK; 3 Medical Retinal, Madras Diabetes Research Foundation & Dr. Mohan’s Diabetes Specialities Centre, Chennai, India; 4 Smt Kanuri Santhamma Centre for Vitreoretinal Diseases, LV Prasad Eye Institute, Hyderabad, India; 5 Department of Vitreo-Retina, Giridhar Eye Institute, Kochi, Kerala India; 6 Department of Retina and Uvea, Sadguru Netra Chikitsalaya, Chitrakoot, Madhya Pradesh India; 7 Department of Vitreo Retinal Services, Shri Ganapati Nethralaya, Jalna, India; 8 Department of Vitreo Retina Surgery, Aditya Jyot Eye Hospital, Mumbai, India; 9 Vision Care & Research Centre, Madhya Pradesh, Bhopal, India; 10 Department of Retina and Vitreous Service, LV Prasad Eye Institute, Bhubaneswar, Odisha India; 11 National University of Singapore, Singapore, Singapore

**Keywords:** Health care, Medical research

## Abstract

To provide the real-world outcomes of people with proliferative diabetic retinopathy (PDR) in India and highlight opportunities for improvement of their disease status and to evaluate their visual acuity (VA) status. A multicenter retrospective study in which ten centers in India with established vitreoretinal services for over 10 years were invited to provide long-term data on PDR. This study population were of Indian nationality. Patients with a diagnosis of type 1 or 2 diabetes with a clinical diagnosis of active PDR in any or both eyes, who had long term follow-up for up to 10 years were included. Baseline data collected included age, sex, duration of diabetes, source of referral and best-corrected visual acuity, diabetic retinopathy status in both eyes. Available follow-up data on VA were collected at 6 months post baseline, 5 years and 10 years within a ± 3 months window. Evaluating the presenting VA of people with PDR, short-term outcomes at 6 months and the incidence of visual impairment (VI) at 5 and 10 years are the main outcome of the study. Data was available for 516, 424 and 455 patients at baseline, 5 years and 10 years respectively. Gender and duration of diabetes did not have statistically significant effect on VI outcomes. Eyes receiving treatment early in the disease course (i.e. baseline VA ≥ 6/12) had significantly better VA outcomes at 10 years versus eyes treated at a later stage (i.e. baseline VA < 6/12) (*p* = <0.0001). On comparing eyes with stable treated PDR and persistent PDR at end of 10 year follow up, a significantly higher percentage of eyes in the stable treated group maintained VA of ≥ 6/12 (55.1% vs. 24.2%) (*p* = < 0.0001), indicating persistent disease activity due to inadequate treatment results in worse VA outcomes. We found no trend in VI or blindness with increasing levels of age at both 5- and 10-year time points (*p* > 0.05). The age standardized incidence for VI was 11.10% (95% CI 8.1, 14.2) and for blindness was found to be 7.7% (95% CI 5.2, 10.3). Our results suggest that despite robust recent clinical trial results showing that pan retinal photocoagulation is an excellent treatment for PDR, people with diabetes in India need to be made aware of annual screening and treatment of their eyes to avoid vision impairment and blindness.

## Introduction

Proliferative diabetic retinopathy (PDR) is a treatable cause of severe visual loss in people with diabetes. If left untreated, most eyes with low risk PDR characterized by mild to moderate retinal or optic disc neovascularization progress to high risk PDR with increasing retinal or disc neovascularization. These eyes remain symptomless until the onset of complications such as vitreous hemorrhage, tractional retinal detachment or diabetic macular edema (DME)^1^. Systematic screening and timely treatment of PDR in countries with established screening programs have resulted in a decrease in the rate of blindness and the incidence of Advanced Diabetic Eye Disease (ADED) over time^2^. Screening for diabetic retinopathy (DR) is still at its infancy in most low and middle-income countries (LMIC)^3^. There is limited literature on the presenting visual acuity (VA) of patients with PDR and their mode of referral to eye care centers in LMIC.

Pan retinal photocoagulation (PRP) has been the standard of care for PDR, with vitreo-retinal surgery advocated for patients with complications^1,4,5^. Recent studies have reported anti-vascular endothelial growth factor (anti-VEGF) therapy as alternative first line treatment for PDR^6–8^. It is unclear, however whether these protocols for treatment can be implemented well across hospitals in LMIC. In countries such as India, where most patients are dependent on out of pocket expenses for their healthcare, the management of diabetic eye disease is influenced by cost of care, lack of screening programs and the lack of public awareness of diabetic eye disease and the need for regular follow-up for ongoing treatment. There is also a wide variation in provision of healthcare in India, with some centers providing world-class services to others that do not have basic facilities or personnel to provide treatment. Therefore, in order to assess the visual outcomes and incidence of visual impairment (VI) in chronic conditions such as PDR in LMIC, it is best to focus on some of the best treatment centers in the country that provide a comprehensive treatment package for PDR and are more likely to have the data to provide the treatment accounts and the prevalence and incidence of VI in people being treated for this condition.

The aims of this study were to evaluate the presenting VA of people with PDR, short-term outcomes at 6 months and the incidence of VI at 5 and 10 years to highlight opportunities for improvement in India and other LMICs.

## Methods

The study protocol was approved by the Institutional Review Board (Ethics committee) at Vision Research Foundation, Sankara Nethralaya, Chennai. As it is a retrospective study, the informed consent was waived by the ethics committee and it adhered to the tenets of the Declaration of Helsinki. Twenty centres in India with established vitreoretinal services for over 10 years were invited to provide long-term data on PDR based on the availability of either electronic patient records or paper registers that enabled retrieval of long-term data. Only 10 centres were able to provide this data.

### Study population

This study population were of Indian nationality from different regions of India. Patients with a diagnosis of type 1 or 2 diabetes with a clinical diagnosis of active PDR in any or both eyes, who presented to these centres in 2008 and had long term follow-up for up to 10 years were included in the study. Clinical practice in India for treatment of PDR involves PRP for low and high risk PDR. Anti-vascular endothelial growth factor (VEGF) injections are given in cases with DME. For advanced PDR appropriate surgical intervention is performed when indicated. The patients could have prior treatment in another hospital before being seen in these centres so the study population consisted of treatment naïve PDR and those with persistent PDR post initial PRP. The patients could also have concomitant DME.

### Study design

This is a multi-centre retrospective cohort study. The patient data were identified either from electronic patient records or manually from registers maintained since 2008 to allow for outcome measurements at 6 months, 5 and 10 years. Consecutive patients who met the inclusion criteria were included in the study.

### Baseline data

Baseline data collected included age, sex, duration of diabetes, source of referral and best-corrected visual acuity (BCVA), DR status in both eyes, presence of DME. PDR status was defined as per the Early Treatment Diabetic Retinopathy Study (ETDRS) classification^9^. Low risk PDR included retinal neovascularization (NVE) < 1.27 mm^2^ (level 61) or disc neovascularization (NVD) < 0.74 mm^2^ and/or NVE ≥ 1.27 mm^2^ (level 65). High risk PDR included NVD < 0.74 mm^2^ and/or NVE ≥ 1.27 mm^2^ and/or vitreous haemorrhage/pre retinal haemorrhage (VH/PRH) ≥ 2.5 mm^2^ (level 71) or NVD ≥ 0.75 mm^2^ and or VH/PRH not obscuring the macula (level 75). Advanced PDR (level 81, 85) was defined as level 75 with VH/PRH obscuring macula. Presence of VH, retinal detachment, fibrovascular proliferation, neovascular glaucoma were also recorded. At final follow up, active/persistent PDR was defined as eyes with new features suggesting reactivation of proliferation or potentially sight threatening complications of fibrous proliferation. Stable treated PDR was defined as eyes with evidence of photocoagulation, regressed neovascularization and absence of features of active disease.

### Follow-up data

Available follow-up data on VA were collected at 6 months post baseline, 5 years and 10 years within a ± 3 months window. The numbers of PRP sessions, cataract surgery, treatment of DME and vitrectomy with or without retinal surgeries were recorded within the first 6 months when most PDR eyes should stabilize if adequate PRP is given and total number of concomitant procedures over 10 years collected to understand the long-term treatment requirements.

### Visual acuity

It is routine practice to perform BCVA using Snellen charts in these centres. However, the optometrists recorded the BCVA measurements in busy clinic settings. Under these circumstances, it is possible that the optometrists had not spend enough time to encourage the patients to read as far as possible. As a result, the BCVA may have been underestimated at times.

### Definition of visual impairment and blindness

There are no universally accepted criteria for VI, blindness (severe VI) or changes in VI. Thus, VI was defined using both the United States (US) and World Health Organisation (WHO) criteria. Incidence of VI applied only to those with no VI at baseline. In the US criteria, no VI was defined as eyes with BCVA 6/12 or better, VI was defined as worse than 6/12 but better than 6/60 Blindness was defined as 6/60 or worse. The WHO criteria defined no VI as 6/18 or better, VI was worse than 6/18 but no worse than or equal to 3/60 and blindness was defined as worse than 3/60. These definitions were also used for both eyes, better-seeing eye and worse seeing eye^10,11^.

### Statistical analyses

Descriptive statistics was used to analyse the baseline characteristics. The visual outcomes at 6 months, 5 and 10 years were analysed to report mean and median outcomes at each time points for all eyes, better eye and worse eye at individual levels. The last observation was not carried forward in this study as it is long-term study. The 5-year and 10-year incidence of monocular VI and blindness in different age groups were calculated as the proportion of the number of new eyes with VI and blindness to the number of eyes with no VI at baseline. Chi square (Cochran Armitage) for trend was used to look at incidence of VI and blindness with increasing age. Multivariable regression was used to analyse the effect of baseline factors on VI and blindness. One way ANOVA was used to compare VA outcomes based on type of referral.

## Results

The baseline characteristics of the study cohort from 10 centres are summarised in (Table 1). Data was available on a total of 519 patients; VA data was available in 516 patients at baseline, 431 patients at 6 months, 424 patients at 5 years and 455 patients at 10 years. In our cohort, gender and duration of diabetes did not have a statistically significant effect on the final VA outcomes.

**Table 1:** Baseline characteristics of the Study Cohort.

Basic characteristics	Study Cohort
Mean age, in years (SD)	52.3(9.2)
Age < 40 years	45/519 (8.7%)
Age 40–59 years	361/519 (69.5%)
Age ≥ 60 years	113/519 (21.8%)
Sex M:F	2.3:1 (362:157)
Mean duration of diabetes in years (SD)	15.9 (9.0)
Median (IQR)	15 (10)
Proportion of eyes with DME	426/1,032 (41.2%)
**Referral source**	
Self referral	198 (31.8%)
Referred by another doctor or hospital	154 (29.7%)
Screening referral	167 (32.2%)
BCVA 6/12 or better both eyes	245/516 (47.5%)
BCVA worse than 6/12 to better than 6/60 (visual impairment in both eyes)	69/516 (13.4%)
BCVA 6/60 or worse in both eyes (blindness)	39/516 (7.6%)
**BCVA in better seeing eye**	
BCVA 6/12 or better	408/516 (79.1%)
BCVA worse than 6/12 to better than 6/60 (visual impairment)	70/516 (13.5%)
BCVA 6/60 or worse (blindness)	38/516 (7.4%)
**BCVA in worse seeing eye**	
BCVA 6/12 or better	245/516 (47.5%)
BCVA worse than 6/12 to better than 6/60 (visual impairment)	111/516 (21.5%)
BCVA 6/60 or worse (blindness)	160/516 (31.0%)
**Fundus picture in both eyes**	
Low Risk PDR (ETDRS 61, 65)	193/516 (37.4%)
**High risk PDR**	
(ETDRS 71, 75)	82/516 (15.9%)
**Advanced PDR**	
(ETDRS 81–85)	41/516 (7.9%)
**Fundus picture in better eye**	
NPDR	40/516 (7.6%)
Low Risk PDR (ETDRS 61, 65)	295/516 (57.1%)
High Risk PDR (ETDRS 71, 75)	140/516 (27.1%)
Advanced PDR (ETDRS 81–85)	41/516 (7.9%)
**Fundus picture in worse eye**	
Low Risk PDR (ETDRS 61, 65)	216/516 (41.8%)
High risk PDR (ETDRS 71, 75)	159/516 (30.8%)
Advanced PDR (ETDRS 81–85)	141/516 (27.3%)
Treatment Naïve eyes at baseline	573/1,032 (55.5%)
**Treatment naïve eyes**	
BCVA 6/12 or better	406/573 (70.8%)
BCVA worse than 6/12 to better than 6/60 (visual impairment)	97/573 (16.9%)
BCVA 6/60 or worse	70/573 (12.2%)
Persistent PDR post initial PRP	390/1,032 eyes (37.8%)
Stable PDR post initial PRP	69/1,032 (6.6%)
Treatment required in both eyes at presentation	439/516 (85.0%)

*SD* standard deviation, *IQR* inter quartile range, *BCVA* best corrected visual acuity, *DME* diabetic macular edema, *PDR* proliferative diabetic retinopathy, *ETDRS* early treatment diabetic retinopathy study.

Using US criterion for VI, the odds ratios (OR) for the age categories (reference < 40 years) were as follows: 1.35 (0.55–3.33), 1.04 (0.44–2.47), 1.02 (0.40–2.59) and 1.63 (0.13–20.36); OR for sex (reference female) was 0.99 (0.61–1.60). Based on WHO criterion for VI, the OR for age were as follows (reference age < 40): 0.97 (0.37–2.51), 0.83 (0.34–2.05), 0.70 (0.25–1.95) and there were no cases for age 70 +, therefore no OR reported for this group. The OR for sex (reference female) was 1.08 (0.62–1.88). For all comparisons, *p* values were not statistically significant at the 5% level.

Although PRP was the main treatment done for all eyes with active PDR, other interventions were also required over 10 years. Eyes with PDR and DME were mainly treated with macular laser although the records on numbers of sessions of PRP or macular laser were incomplete and were not used in any analysis. Among the methods for patient referral, most PDR eyes identified and referred from screening programs were in the early course of disease (VA ≥ 6/12) when compared to self-referral where most patients presented quite late in the disease course (VA < 6/12) (*p* = 0.0002). At 10 years, higher number of eyes diagnosed at via screening ended up maintaining VA ≥ 6/12 than other modes of referral (*p* = 0). (Table 2) shows the proportions of eyes that underwent the interventions at 6 months and 10 years.

**Table 2:** Interventions performed over 6 months and 10 years.

Interventions up to 6 months	Over 6 months	From 6 months to 10 years
PRP only ± macular laser	577/1,032 (55.9%)	875/1,032 (84.8%)
Anti-VEGF	167/1,032 (16.2%)	144/1,032 (14%)
Vitrectomy ± endolaser and retinal surgery	326/1,032 (31.6%)	334/1,032 (32.4%)
Cataract surgery	620/1,032 (60.0%)	No data

*PRP* pan retinal photocoagulation.

The change in visual outcome at 6 months, 5 and 10 years are shown in (Table 3).

**Table 3:** Visual acuity outcomes at each time point.

Visual acuity	Baseline (n = 1,032)	6 months (n = 882)	5 years (n = 848)	10 years (n = 910)
BCVA 6/12 (0.3) or better	653/1,032 (63.3)	555/882 (62.9)	505/848 (59.6)	524/910 (57.6)
BCVA worse than 6/12 but better than 6/60 (1.0) (visual impairment)	181/1,032 (17.5)	169/882 (19.2)	188/848 (22.2)	190/910 (20.9)
BCVA 6/60 or worse (legal blindness)	198/1,032 (19.2)	158/882 (17.9)	155/848 (18.3)	196/910 (21.5)

*BCVA* best corrected visual acuity.

‘n’ at each time point represents the number of eyes for which data was available and therefore is not a longitudinal change of VA in the same observations.

The PDR status of the eyes at 10 years was also analyzed based on their baseline status. Of the 499 eyes with low risk PDR, 306 (77.3%) were stable PDR, 26 (5.2) % progressed to high risk PDR and 36 (7.2%) developed advanced PDR. Out of 299 eyes with baseline high risk PDR, 122 (40.8%) were stable treated PDR, 28 (9.3%) progressed to advanced PDR. Only 36/182 (19.7%) of the eyes with baseline advanced PDR were stable and treated.

Of the 1,032 eyes at baseline, 877 eyes had data on the PDR status at 10 years. All eyes with no PDR at baseline had developed PDR by 10 years. Of the 645 (73.5%) eyes with stable treated PDR at 10 years, 356 (55.1%) had VA of 6/12 or better while 199 (30.8%) and 90 (13.9%) eyes had VA of worse than 6/12 and better than 6/60 and 6/60 or worse respectively. Of the 95 (10.8%) eyes with persistent high-risk PDR at 10 years, 23 (24.2%), 49(51.6%) and 23(24.2%) eyes had a VA of 6/12 or better, worse than 6/12 and better than 6/60 and 6/60 or worse respectively. Almost 3/4th (75.8%) eyes in the persistent PDR group had VA < 6/12 as against 45% in the stable treated group (*p* = <0.0001).

Among the treatment naïve eyes at baseline with early diagnosis in disease course (i.e. VA better than 6/12) and early treatment initiation resulted in 65.7% (267) eyes to maintain VA better than 6/12 at 10 year follow up. Also only 26.1% (106) eyes among these ended up with VA worse than 6/12 at end of 10 years. This was in contrast to treatment naïve eyes at baseline with VA worse than 6/12 i.e. eyes that were diagnosed late and thus treated late in the course of the disease. Only 31.7% (53) eyes were able to achieve and maintain VA better than 6/12, and as high as 46.7% (78) eyes had VA worse than 6/12 at 10 year follow up (*p* < 0.0001).

New onset VI and blindness by 5 years is shown in (Table 4).

**Table 4:** Five-year incidence of best corrected visual impairment and blindness stratified by age at baseline in participants.

Age at baseline (years)	Incidence of visual impairment			Incidence of blindness		
	N	n	% (CI)	N	n	% (CI)
United States Criterion*						
< 40	31	1	3.2 (0.5, 22.2)	35	3	8.6 (2.9, 25.3)
40–49	83	20	24.0 (16.4, 35.3)	94	7	7.4 (3.7, 15.2)
50–59	145	26	17.9 (12.7, 25.4)	179	20	11.2 (7.4, 16.9)
60–69	74	14	18.9 (11.8, 30.3)	82	7	8.5 (4.2, 17.3)
70 +	2	0	NA	3	0	NA
			*p* = 0.51			*p* = 0.82
Crude Overall	335	61	18.2 (14.5, 22.8)	393	37	9.4 (6.9, 12.8)
**WHO Criterion**^**φ**^						
< 40	34	3	8.8 (3.0, 26.0)	36	3	8.3 (2.8, 24.6)
40–49	88	8	9.0 (4.7, 17.6)	99	5	5.0 (2.1, 11.9)
50–59	167	26	15.6 (11.0, 22.2)	184	12	6.5 (3.8, 11.3)
60–69	77	11	14.3 (8.3, 24.7)	87	3	3.4 (1.1, 10.5)
70 +	2	0	NA	3	0	NA
			*p* = 0.24			*p* = 0.82
Crude overall	368	48	13.0 (10.0, 17.0)	409	23	5.6 (3.8, 8.4)

N = number at risk at baseline; n = incident cases; % (CI) = prevalence and 95 percent confidence interval. *p* value calculated using test of trend; Number of observations = 424.

U.S. Criterion* Incidence of visual impairment: Person with Baseline best corrected visual acuity (BCVA) of 6/12 or better in better seeing-eye, and follow-up BCVA worse than 6/12 but better than 6/60 (not including 6/12 or 6/60) in better seeing-eye. Incidence of Blindness: Persons with Baseline BCVA better than 6/60 in the better Seeing Eye and follow-up BCVA 6/60 or worse (including 6/60).

W.H.O. Criterion^φ^ Incidence of visual impairment: Persons with Baseline BCVA 6/18 or better in better seeing-eye and follow-up BCVA worse than 6/18 but better than or equal to 3/60 (not including 6/18 but including 3/60) in the better seeing-eye. Incidence of Blindness: Persons with Baseline BCVA 3/60 or better in better seeing-eye and follow-up BCVA worse 3/60 (not including 3/60) in better seeing-eye.

*NA* no incident cases.

335 eyes with baseline VA of 6/12 or better (based on US criterion) and 368 eyes with VA 6/18 or better (based on WHO criterion) were at risk for vision loss over the study period. 61/335 (18.2%) eyes at baseline worsened from 6/12 or better and 48/368 (13%) worsened from 6/18 or better. Over 75% eyes that worsened from 6/12 and 70% that worsened from 6/18 were aged between 40 and 59 years. Moreover, according to WHO criteria, 409 eyes with VA better than 3/60 at baseline in the better seeing eyes had the potential to become blind by 5 years. By 5 years, 37/393 (9.4%) and 23/409 (5.6%) eyes became blind according to US and WHO criterion respectively.

Over 10 years, 373 eyes with baseline VA of 6/12 or better (based on US criterion) and 405 eyes with VA 6/18 or better (based on WHO criterion) were at risk for vision loss over the study period. Incident VI defined as worsening from 6/12 or better according to US criterion was 66/373 (17.6%). Based on WHO criterion it was 45/405 (11.11%) with majority of these incident cases seen in the age-group 40–59 years (Table 5). Moreover, according to WHO criteria of blindness, 441 eyes with VA better than 3/60 at baseline in the better seeing eye had the potential to become blind by 10 years. By 10 years, 55/425 (12.9%) and 34/441 (7.7%) eyes became blind according to US and WHO criterion respectively.

**Table 5:** Ten-year incidence of best corrected visual impairment and blindness stratified by age at baseline in participants.

Age at baseline (years)	Incidence of visual impairment			Incidence of blindness		
	N	n	% (CI)	N	n	% (CI)
United States Criterion*						
< 40	34	4	11.8 (4.7, 29.5)	40	6	15 (7.2, 31.4)
40–49	91	17	18.7 (12.2, 28.7)	103	15	14.6 (7.2, 18.6)
50–59	164	28	17.1 (12.2, 23.9)	191	26	13.6 (9.5, 19.5)
60–69	81	16	19.8 (13.7, 30.6)	87	8	9.2 (4.8, 17.8)
70 +	3	1	33.3 (6.7, 165.1)	4	0	NA
			*p* = 0.39			*p* = 0.22
Crude overall	373	66	17.7 (14.2, 22.0)	425	55	12.9 (9.5, 15.6)
**WHO Criterion**^φ^						
< 40	38	4	10.5 (4.2, 26.6)	41	3	7.3 (2.5, 21.8)
40–49	98	13	13.3 (8.0, 22.0)	107	8	7.5 (3.8, 14.6)
50–59	181	21	11.6 (7.8, 17.3)	196	16	8.2 (5.1, 13.0)
60–69	85	7	8.2 (4.0, 16.7)	93	7	7.5 (3.7, 15.3)
70 +	3	0	NA	4	0	NA
			*p* = 0.40			*p* = 0.96
Crude overall	405	45	11.1 (8.4, 14.6)	441	34	7.7 (5.6, 10.6)

N = number at risk at baseline; n = incident cases; % (CI) = prevalence and 95 percent confidence interval; *p* value calculated using test of trend; Number of observations = 455.

U.S. Criterion*. Incidence of visual impairment: Person with Baseline best corrected visual acuity (BCVA) of 6/12 or better in better seeing-eye, and follow-up BCVA worse than 6/12 but better than 6/60 (not including 6/12 or 6/60) in better seeing-eye. Incidence of Blindness: Persons with Baseline BCVA better than 6/60 in the better Seeing Eye and follow-up BCVA 6/60 or worse (including 6/60).

W.H.O. Criterion^φ^. Incidence of visual impairment: Incidence of visual impairment: Persons with Baseline BCVA 6/18 or better in better seeing-eye and follow-up BCVA worse than 6/18 but better than or equal to 3/60 (not including 6/18 but including 3/60) in the better seeing-eye. Incidence of Blindness: Persons with Baseline BCVA 3/60 or better in better seeing-eye and follow-up BCVA worse 3/60 (not including 3/60) in better seeing-eye.

*NA* No incident cases.

The age standardized incidence (standardized to the study population) of VI was 17.7% (95% CI 13.9, 21.6) using US criterion and 11.10 (95% CI 8.1, 14.2) based on WHO criterion at 10 years. The age standardized incidence (standardized to study population) for blindness was 12.9 (95% CI 9.7, 16.1) based on US criterion and 7.7% (95% CI 5.2, 10.3) with WHO criterion at 10 years. The age standardized incidence (standardized to India census population 2001) of VI was 14.2 (95% CI 7.1, 21.3) and 9.3 (95% CI 3.6, 14.9) using US and WHO criterion respectively. The age standardized incidence (standardized to India census population 2001) of blindness was 14.6 (95% CI 7.9, 21.4) based on US and 14.6 (95% CI 7.7, 21.5) using WHO criterion at 10 years. Additionally, we found no trend in VI or blindness with increasing levels of age at both 5- and 10-year time points.

## Discussion

Contemporary data from clinical trials on PDR patients conducted in high-income countries show that PRP remains an ideal treatment option for PDR with good short and long-term visual outcomes despite a 40% drop-out of patients by 5 years^6–8^. However, our 10-year study results from India reveal a few important points that may be of relevance to all LMIC.

Firstly, the baseline characteristics show that although the demographic features of these patients are similar to those reported from Western countries, only a third of patients are referred from DR screening programs. Inadequate screening contributes to poor presenting vision^12^. Nearly 20% of the study cohort presented with VI or blindness in the better-seeing eye. However, the patients diagnosed through screening had better presenting VA and final visual outcomes, reinforcing the importance of screening programs.

Moreover, at presentation, approximately a third of patients presented with high risk PDR and 8% of the better seeing eye had ADED explaining why vitreoretinal surgery was required in a third of individuals in the first 6 months. This point further highlights the late presentation of a significant number of patients for treatment. Presenting VA and severity of PDR are both predictors of visual outcome^13^. Therefore, it is imperative that policies are in place for systematic screening and care pathways be designed for timely treatment and follow-up of this high-risk group. The challenges include the costs of laser devices and expertise required at the treatment centers.

Although our baseline data is obtained in 2008, the reports from the Vision Loss Expert Group of the Global Burden of Disease Study show that the prevalence of blindness due to DR has not decreased from 1990 to projected figures in 2020^14^. Whilst the prevalence of PDR in people with diabetes is about 3% in India, PDR and its complications are the most common cause of DR related blindness. As PDR is initially asymptomatic, another barrier to treatment is the lack of public and patient awareness of the need for timely treatment. Therefore, more patient education and advocacy programs have to be initiated to ensure these changes.

After initial PRP, most patients will require fill-in sessions. In Protocol S, 38% required further laser in the first 6 months^6^. In our study, 1,070 interventions for PDR were required by 6 months and a further 283 procedures were required over the 10 years indicating that PRP is not a one off procedure and that the patients need to be monitored regularly over a prolonged period^15^. In addition, only 73.5% had stable treated PDR. Over 10 years, 12% of eyes progressed to high risk PDR and a further 10% high risk PDR to advanced eye disease and 10% remained high risk, substantiating the fact that PDR eyes need to monitored closely over 10 years and treatment given as required.

This study also shows that despite PRP, nearly 50% of the patients had VI at 10 years, suggesting that patients may not be lasered sufficiently or may be monitored less frequently than required.

When we consider incident cases of VI, we found that on average, 17% develop VI 10% become blind by 5 years and these figures worsen marginally by 10 years. In contrast, only 9% and 6% of participants showed 10 or 15 letters worsening after 5 years in Protocol S^8^.

Our study results are similar to those reported from short term studies in other LMIC highlighting the challenges in the management of PDR in resource constrained countries. A population based study also revealed similar prevalence of 6.3% of blindness due to DR and reinforced that baseline VA is an important predictor of visual outcome in PDR^16^. Other hospital based studies from LMIC show similar prevalence of clinic based blindness due to PDR^17^.

Monitoring of the systemic parameters such as glycemic control, blood pressure control and the renal parameters during the follow-up of patients treated for PDR would be invaluable for sustaining improvement in visual outcomes and reducing the burden of VI^17^.

As PDR is the most common cause of blindness, the resource requirements for these individuals are higher. Around 60% require cataract surgery and a third require vitreo-retinal surgery. There are approximately 3 million people with PDR in India and there are less than 1,000 practicing vitreo-retinal surgeons equating to one surgeon for every 3,000 patients. With the lack of trained human resources for vitreo-retinal surgery, it is obvious that the LMIC countries are in a vicious cycle of limited resources, lack of screening, poor presenting VA and need for vitreoretinal surgery. The only opportunity to break this cycle is to implement screening programs, strengthen the primary care system to control the risk factors of retinopathy and improve health seeking behaviors of our patients by increasing their awareness of the need for screening and frequent monitoring of their eyes.

The strength of this study is it provides both short- and long-term outcomes of PDR in a LMIC. There is a paucity of these types of studies globally. The study highlights an urgent need for quality improvement of the care we provide patients with diabetes at high-risk of visual loss. The study incorporated data from 10 centers in India so the results are generalizable and represents the highest quality of care provided in India.

Limitations of this study include the retrospective nature of the study but the clinics have the BCVA measured as a routine so the quality of data is good. The possibility of under-reporting cannot be excluded, nor can its magnitude, if present, be determined. However, the outcomes resemble those from other studies done in LMIC^18,19^. It is possible that severely ill people are under-represented in this dataset. Selection bias may also influence the outcome of the study. Only patients who were followed up for up to 10 years are included in this study. As these patients should ideally have received the best care compared to those who were lost to follow-up, the study results may have underestimated the prevalent and incident cases of VI and blindness. However, the magnitude of VI in this study cohort indicates the need for implementation of national improvement programs. A further recommendation is the need for electronic medical records to ensure the roll out of frequent service evaluations and quality improvement programs.

In summary, our results reinforce the need for improved public awareness of sight threatening complications of diabetes, systematic DR screening and prompt treatment of PDR to reduce the magnitude of vision impairment and blindness in people with diabetes.

## Data Availability

The datasets generated during and/or analysed during the current study are not publicly available, as it is against the organization/hospital policy. But are available from the corresponding author on reasonable request.

## Abbreviations

PDR: Proliferative diabetic retinopathy
DME: Diabetic macular edema
ADED: Advanced Diabetic Eye Disease
LMIC: Low and middle-income countries
PRP: Panretinal photocoagulation
NVE: Retinal neovascularization
NVD: Disc neovascularization
BCVA: Best-corrected visual acuity
VI: Visual impairment
VA: Visual acuity
DR: Diabetic retinopathy
Anti-VEGF: Anti-vascular endothelial growth factor
ETDRS: Early Treatment Diabetic Retinopathy Study
VH: Vitreous haemorrhage
PRH: Pre retinal haemorrhage
WHO: World Health Organisation
US: United States
